# Integrating technology-assisted behavioral management into a summer obesity prevention intervention program: a pilot controlled trial

**DOI:** 10.3389/fphys.2026.1914161

**Published:** 2026-09-16

**Authors:** Qiaoyin Tan, Paul Son, Yuxin Nie, Pengfei Yang, Amanda E. Staiano, Fahui Wang, David C. Shepherd, Katie Queen, Stewart Gordon, Senlin Chen

**Affiliations:** 1 School of Kinesiology, Louisiana State University, Baton Rouge, LA, United States; 2 Pennington Biomedical Research Center, Baton Rouge, LA, United States; 3 Department of Geography, University of Hong Kong, Hong Kong, Hong Kong SAR, China; 4 Division of Computer Science & Engineering, Louisiana State University, Baton Rouge, LA, United States; 5 Our Lady of the Lake Regional Medical Center, Franciscan Missionaries of Our Lady Health Systems, Baton Rouge, LA, United States

**Keywords:** adolescents, artificial intelligence, behavior change, digital health, physical activity, summer camp

## Abstract

**Background:**

Adolescents commonly experience weight gain during summer months due to less structured daily schedules.

**Objectives:**

The pilot study aimed to assess (1) acceptability of the AI-assisted *ProudMe Tech* mobile app integrated within an 8-week long health promotion summer intervention, (2) usage of three technology-assisted behavioral management components (i.e., mobile app, accelerometer and Fitbit-based activity monitoring), and (3) preliminary impact of this summer intervention on anthropometric, behavioral, and psychosocial outcomes among adolescents.

**Methods:**

Twenty-five participants (ages 11–14) were allocated to the intervention group (IG; *n =* 14, one withdrew after Week 1) or control group (CG; *n =* 12). IG attended a 10-day in-person camp (Monday-Friday for two weeks, 5 hours per camp day) consisting of physical education lessons, nutrition messaging, campus walks, and technology-assisted behavioral management. The campers completed an acceptability questionnaire twice during camp (once per week) and wore both Fitbit and ActiGraph accelerometers during camp hours. Out of the 2 weeks camp, participants continued using the *ProudMe Tech* mobile app and wearing Fitbit with parental engagement for 6 weeks. The CG attended a weekly virtual computer coding program over 8 weeks. Anthropometrics (i.e., weight, height, waist circumference), health behaviors (physical activity [PA], screen time, fruit/vegetable intake), health knowledge, and activity self-efficacy were measured at baseline and week 8 for all participants, while campers’ anthropometrics were also measured at end of the camp (day 10).

**Results:**

The *ProudMe Tech* mobile app and intervention demonstrated modest acceptability, with total acceptability percentage scores increasing modestly from Week 1 to Week 2 (54.4 ± 21.8% to 58.3 ± 20.3%; Cohen’s *d* = 0.43). Technology-use data showed that the digital components could be implemented during the structured camp period, although app engagement declined during the post-camp extension period. Campers averaged 121.3 min/day of accelerometer-assessed Moderate-to-Vigorous Physical Activity (MVPA) during the 10-day camp, and 851 mobile app entries were recorded across the 8-week intervention, although app engagement declined after the in-person camp period. Among participants with usable Fitbit data, Fitbit-recorded PA was higher on weekdays than weekends (166.2 vs. 135.6 min/day, *p* = .039). Secondary exploratory outcomes showed mixed and mostly non-significant patterns. The IG showed descriptively smaller weight gain and a more stable BMI pattern than the CG, but group × time interactions for anthropometric outcomes were not statistically significant. Campers’ waist circumference decreased from baseline to the end of camp but rebounded by post-intervention. Self-reported health behaviors and psychosocial outcomes showed no significant group × time interaction effects.

**Conclusions:**

This pilot study showed that technology-assisted behavioral management had modest acceptability and usage, and the summer intervention made mixed impact on the campers’ anthropometric, behavioral, and psychosocial outcomes. Larger future randomized trials are needed to evaluate the efficacy of technology-assisted summer adolescent obesity prevention interventions through longer camp exposure and greater parental engagement.

## 1 Introduction

Summer months represent a critical period for accelerated weight gain and unhealthy lifestyle behaviors among children and adolescents (Guerrero-Magaña et al., 2025; Tanskey et al., 2018). Many adolescents often exhibit reduced physical activity (PA), increased screen time, irregular sleep patterns, and unhealthy eating during summer months (Dambel et al., 2025). The *structured day hypothesis* provides a theoretical explanation for this seasonal pattern, suggesting that the regulated routines of the school environment, characterized by consistent scheduling, adult supervision, and limited discretionary choices, help constrain obesogenic behaviors (Brazendale et al., 2017). In contrast, the absence of structure during summer months creates conditions under which unhealthy behaviors may proliferate, thereby elevating the risk for childhood obesity (Zosel et al., 2022).

Summer health promotion programs may offer adolescents structured opportunities to engage in PA, nutrition education, and other health-enhancing experiences (Larsen et al., 2016; Zeraattalab-Motlagh et al., 2025). However, traditional summer camps often come with challenges. Many of these camps, for example, are costly and require reliable private transportation, which are less accessible for lower-income families (Franckle et al., 2014; Wang and Lim, 2012). Prior multi-component summer interventions showed positive effects on PA, self-esteem, and social skills (Moreno et al., 2017; Tan et al., 2024). More recently, a randomized clinical trial specifically examined access to a free summer day camp and found that children from low-income households showed improved moderate-to-vigorous physical activity (MVPA) and reduced sedentary and screen time compared with summer-as-usual controls (Beets et al., 2025). Yet the evidence remains mixed, with some summer intervention studies reporting little to no impact on anthropometric or behavioral outcomes (Evans et al., 2020; Larsen et al., 2014). These challenges suggest that summer programs may benefit from additional strategies that extend and reinforce support for effective behavioral management.

One promising strategy is the integration of technology into summer health promotion programs. Mobile apps and wearable devices can extend the reach of these programs by reinforcing core behavior change strategies such as goal setting, self-monitoring (McLoughlin et al., 2019; Pyky et al., 2017), and self-reflection (Sousa Basto and Ferreira, 2025). In addition, wearable self-monitoring tools, such as Fitbit devices (Kiss et al., 2024), can also increase self-awareness of daily health behaviors and vital signs leading to timely behavioral regulation (Au et al., 2024). When integrated into summer health promotion programs (in-person, virtual, or hybrid), these technologies may strengthen behavior change during program hours and provide continuity of support at home and beyond (Tripicchio et al., 2023). Additionally, technological tools may help bridge the transition from supervised programming to independent self-management. Such integration is especially relevant for obesity prevention interventions that target multiple behaviors such as PA, diet, screen time, and/or sleep. A hybrid model that combines an in-person health promotion camp with a technology-supported extension may provide a more scalable approach to sustain the continuity of behavioral management in young adolescents.

Within the broader technology-assisted approach, artificial intelligence (AI) can support behavioral management by generating real-time, structured, and personalized responses to adolescents’ self-monitoring entries (Chen, 2024; Kerth et al., 2025). AI feedback may help adolescents set goals, track behaviors, reflect on progress, and provide behavioral counseling. However, the potential value of integrating these technological tools into summer programs depends not only on their availability, but also on whether adolescents would find them acceptable and usable. Therefore, user acceptability and usage of these technological integrations into summer programs (e.g., camps) are important early feasibility indicators (Bowen et al., 2009) before evaluating the long-term effectiveness of these interventions.

Project *ProudMe* is a multi-component obesity prevention intervention initiative designed to reduce adolescents’ risk for obesity (Nie et al., 2025). The *ProudMe* intervention was originally developed for school-based implementation. Key components including *ProudMe* PE and *ProudMe Tech* were recently adapted for summer delivery in the present study. *ProudMe* PE was altered to add fitness education activities using high intensity interval training (HIIT) in addition to adding ultimate frisbee instruction, in an effort to further improve PA for campers. *ProudMe Tech* is a web-based AI-assisted behavioral management app (Tan et al., 2026). *ProudMe Tech* was altered to develop a mobile app, in an effort to make the technological portion more user-friendly.

This pilot-controlled trial was designed to evaluate *ProudMe* Summer intervention, which integrated an AI-assisted behavioral management app (i.e., *ProudMe Tech*) and technology-assisted self-monitoring, along with other components focused on obesity prevention. Specifically, the study aimed to assess (1) acceptability of the AI-assisted *ProudMe Tech* mobile App, (2) usage of three technology components: *ProudMe Tech* mobile app, accelerometer-based activity monitoring, Fitbit-supported self-monitoring, and (3) preliminary impact of the summer intervention on anthropometric, behavioral, and psychosocial outcomes among adolescents.

## 2 Materials and methods

### 2.1 Study design

This study employed a pilot-controlled trial design with two parallel groups. The intervention group (IG) participated in the *ProudMe* Camp over two weeks (Monday-Friday for 10 days total), followed by a 6-week technology supported extension period in the summer of 2025. The control group (CG) engaged in a weekly virtual computer coding program for 8 weeks. The CG program served as a comparison activity and did not include obesity prevention content, physical activity programming, or technology-assisted behavioral management. Because participants’ availability differed for the in-person camp and virtual coding program, group allocation was based on participant availability rather than random assignment. The study protocol was approved by the Louisiana State University Institutional Review Board (IRBAM-25-0015). Written informed consent from parents/guardians and child assent from participants were obtained before participation. This pilot non-randomized controlled trial was not prospectively registered.

### 2.2 Participants

A total of 26 adolescents voluntarily signed up for the study via parent registration. Because this was a non-randomized pilot-controlled trial, group assignment was based on participants’ availability to attend the in-person camp schedule, summarized in **Figure 1**. Fourteen participants were assigned to the IG and 12 to CG (total of 26), and 25 participants completed the post assessments. Eligibility criteria included: (a) being between 11 and 14 years of age; (b) residing within 10 miles of the university campus specifically in a community with moderate to high level of area deprivation, as indicated by neighborhood-level Area Deprivation Index (ADI) rankings; (c) having access to a personal mobile device for at least 15 minutes per day; (d) having reliable transportation to attend the camp on the designated days.

**Figure 1:**
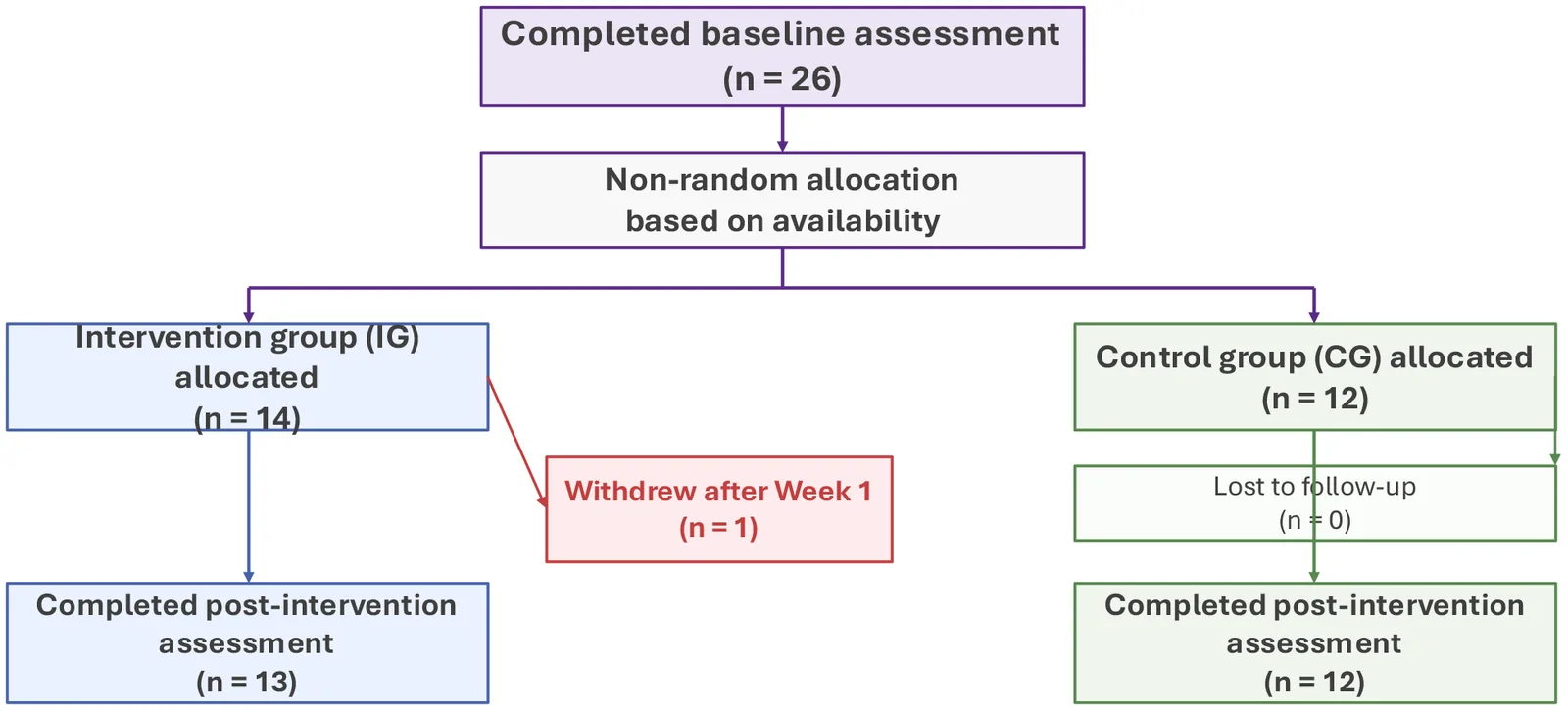
Participant flow diagram.

Participants were recruited through parent-facing flyers and electronic announcements distributed through social media. Recruitment materials described the study as an 8-week summer health promotion research program for adolescents that included either the in-person summer camp or a weekly virtual coding comparison program. Interested parents completed an online registration/screening form before eligibility was confirmed.

### 2.3 Intervention

The *ProudMe* intervention was structured as a summer obesity prevention program that integrated in-person camp activities over two weeks (Monday-Friday for 10 days total) followed by AI-assisted behavioral management through *ProudMe Tech*, ActiGraph-assessed PA, Fitbit-supported self-monitoring, and parent communication for six weeks beyond the camp. During the in-person camp, participants received daily physical education lessons taught by a licensed and state-certified teacher, nutrition messaging over the snack break, goal-setting instruction, free lunch and campus walks. These activities were designed to provide summer structure and support adolescents’ behavioral management for obesity prevention. A trained camp counselor provided instruction about behavioral management and facilitated the campers’ interaction with the mobile app during camp sessions. Participants had mobile device access eligibility so they can continue using the app outside camp during the 6-week extension period. The campers were prompted to set specific, measurable, achievable, realistic/relevant, timely (SMART) goals (Chen, 2024); record their daily behaviors of PA, diet, screen time, and sleep; reflect on their behaviors; and receive AI-generated personalized feedback.

#### 2.3.1 ProudMe Tech

*ProudMe Tech* was a mobile AI-assisted behavioral management application adapted from the original web-based platform (Tan et al., 2026). *ProudMe Tech* used *a React.js* front end, a *Node.js/Express* back end, and a *MongoDB* database, with the ChatGPT-4o API accessed through the server. The mobile app was created by an interdisciplinary LSU team of faculty, staff, and students. The mobile app allowed adolescents to enter daily behavioral goals and outcomes, write brief reflections, and receive immediate feedback based on their self-monitoring entries. *ProudMe Tech* was integrated with a ChatGPT- 4o API based generative AI model to generate real-time, structured responses tailored to participants’ reported behaviors and reflections. The AI-generated feedback was designed to provide encouragement, reinforce goal progress, support behavior reflection, and offer behavioral counseling related to PA, diet, screen time, and sleep. For example, one participant set a screen-time goal of 120 minutes but reported 302 minutes and reflected, “I spent a lot of time on YouTube yesterday. I also wanted to finish my daily quest, so I practiced for an hour to finish it.” The app immediately generated personalized feedback, “It’s great that you’ve reflected on your screen time. However, 302 minutes is significantly above the recommended value. I encourage you to set your goal below 120 minutes and find alternatives, like reading or outdoor activities, to reduce your screen time further. Keep pushing yourself!”.

The system instruction directed the GPT-4o model to generate a brief response corresponding to the level of goal attainment, including positive reinforcement when a goal was met, an educational prompt when a goal was partially met, or a constructive suggestion when goal attainment was low. Model responses were limited to 75 tokens, with the temperature set at 0.9. A 75-token limit corresponds to approximately 50–60 words, while the temperature parameter controls response variability, with 0.9 allowing some variation in wording. Each generated response was processed through the OpenAI Moderation API before being displayed to the participant. The OpenAI API key was stored on the remote server and was not accessible to participants. The model avoided providing medical diagnoses, treatment recommendations, prescriptive weight-loss advice, extreme dietary restrictions, or stigmatizing language. AI-generated responses were reviewed *post hoc* by the research team after completion of the intervention as part of the research analysis. No contraindicated or otherwise unsafe responses were identified during this *post hoc* review. All generated messages were retained in the study database and subsequently examined as part of the research analysis. Therefore, automated content moderation constituted the primary safety mechanism during this pilot implementation.

#### 2.3.2 Parent communication

Parent communication, including virtual parent seminars, reminders, and healthy living resources, was delivered during the in-person camp and the 6-week technology-supported extension period to reinforce obesity prevention messages and behavioral management support at home. Parent-facing materials were delivered on four occasions: week 1,2,4,6. Week 1 materials introduced *ProudMe* and provided USDA guidance on being a healthy role model for children (U.S. Department of Agriculture et al., 2020); The Week 2 materials focused on portion size, healthy eating, and MyPlate recommendations (U.S. Department of Agriculture et al., 2020). The Week 4 materials addressed hidden sugar and screen-time management, whereas the Week 6 communication provided child weight-management resources (Control & Prevention, 2026). These communications were delivered via parent emails as virtual videos and written materials. During week 2-8, families were encouraged to support participants with continued use of *ProudMe Tech* 3–5 times per week and daily Fitbit wear.

Participants were also provided with Fitbit Charge 6 devices to support PA awareness and self-monitoring throughout the *ProudMe* Summer intervention. ActiGraph GT3X+ accelerometers were used in two ways: first, to assess PA outcomes at pre- and post-intervention assessments for both groups, and second, to objectively monitor IG participants’ PA only during the 5-hour in-person camp sessions across the 10 camp days. After the in-person camp ended, participants were encouraged to continue using the *ProudMe Tech* mobile app and wearing Fitbit during the 6-week technology-supported extension period.

### 2.4 Measures

#### 2.4.1 Implementation and technology-use measures

##### 2.4.1.1 Camp intervention fidelity

Teachers’ daily logs were used to monitor intervention fidelity of the physical education lessons during the in-person camp. For each planned lesson activity, teachers documented whether the session was delivered as planned or modified, as well as participants’ focus and enjoyment levels. These logs were used to describe implementation completion, practical adaptations, and participant engagement during the two-hour per day physical education within the camp. Attendance was recorded daily and the intervention was observed by the head researcher to ensure full fidelity.

##### 2.4.1.2 *ProudMe Tech* mobile app acceptability

Acceptability of the *ProudMe Tech* mobile app was assessed using the 24-item *ProudMe app* perceived-attribute questionnaire modified from Atkinson’s questionnaire for measuring perceived attributes of eHealth innovations (Atkinson, 2007). The ProudMe app perceived-attribute questionnaire was adapted from Atkinson’s 30-item perceived-attribute instrument. The 24 retained items represented five domains: relative advantage (8 items), simplicity (5 items), trialability (4 items), observability (3 items), and translatability (4 items). Example items included “*ProudMe* APP is better than using workbooks or paper-and-pencil tests for learning about health behaviors” for relative advantage, “I had no difficulty understanding how to get around in *ProudMe* APP” for simplicity, and “Other students seemed interested in *ProudMe* APP when they saw me using it” for observability. Participants rated each item on a 6-point Likert scale ranging from 1 (strongly agree) to 6 (strongly disagree). Because all retained items were positively worded, lower raw scores indicated more favorable perceptions of the app, and no reverse coding was required. For each domain, the raw scores of the corresponding items were averaged to calculate a domain mean. A total raw mean was calculated by averaging all 24 retained items. Scores were converted to percentage scores using the formula (1 − raw mean/6) × 100, with higher percentage scores indicating greater perceived acceptability. Participants completed the questionnaire on day 4 and day 9 of camp.

##### 2.4.1.3 Technology usage and engagement

Usage and engagement with the technology-assisted behavioral management components were captured through camp-hour ActiGraph GT3X+ accelerometer wear on the waist, Fitbit tracking on the wrist, and *ProudMe Tech* mobile app engagement. For the camp-hour assessment, IG participants wore hip-worn ActiGraph GT3X+ accelerometers only during the 5-hour in-person camp sessions between 8:00 AM and 1:00 PM across the 10 camp days; these data were used to describe PA levels during the structured camp setting, including MVPA, light PA, and sedentary behavior. Separately, ActiGraph accelerometers were also used at pre- and post-intervention assessments for both groups as a preliminary PA outcome measure. Fitbit data were used to describe daily PA patterns on weekdays and weekend days across the 8-week intervention period. Weekly mobile app journal entries were processed and quantified to describe engagement during the structured camp and post-camp extension periods.

#### 2.4.2 Preliminary health related outcome measures

##### 2.4.2.1 Anthropometric outcomes

Participants’ height, weight and waist circumference were measured using the Seca 777 physician’s scales and Seca 201 tension constant tape measure (Seca Corporation, California, USA), respectively. Body mass index (BMI; kg/m²) was calculated from measured height and weight. Weight-status categories were determined according to CDC age and sex growth charts (Hales et al., 2022). Anthropometric outcomes were measured at pre- and post-intervention for both groups. For IG, anthropometrics was additionally measured at mid-intervention on the final in-person camp day.

##### 2.4.2.2 Health behaviors

Health behaviors were assessed using self-reported questionnaires at pre- and post-intervention. Participants completed survey items that captured self-reported PA, fruit/vegetable intake, and screen time behaviors (Miller et al., 2020). Specifically, participants reported the number of days they were physically active for at least 60 minutes in the past week and the frequency of leisure-time MVPA, using questions adopted from the National Health and Nutrition Examination Survey (NHANES) (National Center for Health Statistics, 2024), the amount of time spent in various types of screen time behaviors (e.g., video games, social media, watching shows or movies, and other screentime activities) using a validated survey (Twenge and Campbell, 2018), and the number of servings of fruits and vegetables consumed per day (Hoy et al., 2020). ActiGraph *GT3X*+ accelerometers (ActiGraph LLC, Pensacola, FL, USA) objectively assessed PA by intensity using established accelerometer cut-points (Freedson et al., 2005).

##### 2.4.2.3 Psychosocial outcomes

PA self-efficacy was assessed using an 8-item adolescent PA self-efficacy scale from the *PhenX Toolkit physical activity self-efficacy adolescent* protocol (PhenX Toolkit, 2026). The items assessed adolescents’ confidence in being physically active during free time under common barriers, such as “I can be physically active during my free time on most days”. Participants rated each item on a 5-point Likert-type scale ranging from 1 (*disagree a lot*) to 5 (*agree a lot*). Item scores were summed to produce a total score ranging from 8 to 40, with higher scores indicating greater PA self-efficacy. Internal consistency was assessed using Cronbach’s alpha for the 8-item PA self-efficacy scale (Cronbach, 1951) with coefficients of.83 at baseline and.88 at post-intervention.

##### 2.4.2.4 Health-related fitness knowledge

Health-related fitness knowledge was assessed using 29 multiple-choice items adapted from the PE Metrics Standards 3 and 4 health-related fitness knowledge assessment (Kern et al., 2020). Items covered physically active lifestyles, health-related fitness components, exercise intensity, workout planning, aerobic fitness, muscular strength and endurance, flexibility, warm-up and cool-down, and body composition. For example, items required participants to identify components of health-related fitness and appropriate principles of exercise prescription. Each correct response was assigned one point, whereas incorrect or unanswered items were assigned zero points. The summed score ranged from 0 to 29, with higher scores indicating greater health-related fitness knowledge. Internal consistency was assessed using Cronbach’s alpha. For the dichotomously scored knowledge items, internal consistency was assessed using the Kuder–Richardson Formula 20 (Kuder and Richardson, 1937), with coefficients of.77 at baseline and.57 at post-intervention.

### 2.5 Data collection procedures

**Table 1** shows the data collection procedures. Data were collected at three assessment time points: baseline/pre-intervention (Week 0), post-camp (Week 2; day 10 of the in-person camp; IG only), and post-intervention (Week 8). All data collectors received standard human research ethics training and additional research training prior to the start of the project. Pre- and post-intervention assessments for anthropometrics, self-reported health behaviors, accelerometer-assessed PA, and psychosocial outcomes were administered in the research lab by trained data collectors on our university campus. Mid-intervention assessments were conducted at the end of the two-week in-person camp for the IG (day 10). The campers were asked to wear their personalized ActiGraph accelerometers on their right waist during the 5-hour camp time between 8am and 1pm daily. On days 4 and 9 of the in-person camp, the campers completed the *ProudMe Tech App* acceptability questionnaire. *ProudMe Tech* mobile app usage data, including *ProudMe Tech* mobile app entries, were saved and recorded. Fitbit data were collected throughout the intervention period to describe daily PA patterns. These technology data from the mobile app, Fitbit, and accelerometers were downloaded to a local drive for subsequent processing.

**Table 1:** Study procedures and assessment timeline.

Time	Intervention group	Control group	Data collection
Week 0	Group allocation	Group allocation	Anthropometrics, ActiGraph, health behaviors, PA self-efficacy, fitness knowledge
Weeks 1–2	10-day in-person ProudMe Camp; app use; Fitbit wear; camp-hour ActiGraph	Weekly virtual coding program	IG app acceptability on camp Days 4 and 9*; ProudMe Tech* entries; Fitbit activity records; camp-hour ActiGraph data
End of Week 2	End of in-person camp	Continued coding program	IG anthropometrics only
Weeks 3–8	Continued ProudMe Tech use and Fitbit wear; parent communications in Weeks 4 and 6	Continued weekly coding program	IG’s App and Fitbit usage data
Week 9	Post-intervention assessment	Post-intervention assessment	Anthropometrics, ActiGraph, health behaviors, PA self-efficacy, fitness knowledge

### 2.6 Data processing and analysis

Analyses were conducted to describe implementation patterns, technology usage, and preliminary outcome changes appropriate for a pilot-controlled trial. Descriptive statistics (*M*, *SD*, %, *N*, etc.) were used to summarize participant characteristics, intervention fidelity, *ProudMe Tech* mobile app acceptability, mobile app engagement, camp-hour ActiGraph assessed PA, Fitbit activity summaries, and other implementation and technology-use measures.

ActiGraph data were processed separately for two purposes. First, camp-hour ActiGraph data were used to describe IG participants’ PA during the structured in-person camp setting. These data reflected only the 5-hour camp period between 8:00 AM and 1:00 PM across the 10 camp days, not full-day wear. Camp-hour ActiGraph outcomes were summarized descriptively as time in MVPA, light PA, and sedentary behavior and as percentages of the 5-hour camp period. Second, pre- and post-intervention ActiGraph data were used as preliminary PA outcome measures for both groups. For these pre- and post-intervention accelerometer assessments, a valid day was defined as at least 10 hours of wear time. For Fitbit data, no minimum daily wear-time threshold was applied. A reported day was defined as any exported day with non-zero recorded activity; days with no recorded activity were treated as missing.

The accelerometer data (15-s epoch) were processed using the critical cut-point standard to estimate metabolic equivalent (MET) (Freedson et al., 2005). The MET rate was calculated using the following formula: MET = 2.757 + (0.0015 * CPM) - (0.08957 * age) - (0.000038 * CPM * age), where CPM refers to count per minute. We categorized activity data into MVPA (≥ 3 METs), light PA (LPA; 1.5–2.99 METs), and sedentary behavior (SB; <1.5 METs) and calculated the time (in minutes) and time percentage for subsequent data analysis.

Statistical analyses were conducted using R statistical software (R version 4.5.0). All available baseline data were retained for descriptive analyses. For pre–post and group × time analyses, participants were included only when they had the data required for the corresponding outcome and assessment time points; participants with incomplete data for a specific analysis were excluded from that analysis. Therefore, sample sizes varied across outcomes (e.g. Accelerometer and Fitbit records) and are reported in the corresponding tables and figure legends. Exploratory inferential analyses were conducted for preliminary health-related outcomes. Group-by-time comparisons for anthropometric, pre/post ActiGraph-assessed PA, self-reported behavioral, and psychosocial outcomes were conducted using repeated-measures analyses and mixed ANOVAs, with α = 0.05 for significance testing. *Cohen’s d* was calculated where applicable describe within-group pre-to-post anthropometric changes and between-group differences in change scores. Given the exploratory nature of this pilot study, no formal adjustment for multiple comparisons was applied. All reported p values are therefore nominal and unadjusted. Because multiple pre–post and group × time tests were conducted, the inferential findings were interpreted cautiously, with emphasis placed on effect sizes, overall patterns, and hypothesis generation rather than confirmatory evidence of intervention effectiveness.

## 3 Results

### 3.1 Participant characteristics and fidelity check

As shown in **Table 2**, baseline characteristics include all 26 participants who completed the baseline assessment. One IG participant withdrew after Week 1 but was retained in baseline descriptive analyses. the sample was composed of 64% male participants, with the majority identifying as Black/African American (*n =* 13) or Asian Americans (*n =* 9). *IG* had 3 participants with obesity, 2 overweight; while CG had 2 participants with obesity and 2 overweights. The groups differed notably in racial composition. The CG included primarily Asian American participants, whereas the IG included primarily Black/African American participants. These differences should be considered when interpreting between-group comparisons.

**Table 2:** Participant demographic characteristics.

Variables	Categories	*N*	CG	IG
Age	11	10	3	7
	12	8	5	3
	13	6	3	3
	14	2	1	1
Sex	Male	17	7	10
	Female	9	5	4
Race	Black/African American	13	4	9
	Asian American	9	8	1
	White American	3	0	3
	Hispanic American	1	0	1
Weight Status	Obesity	5	2	3
	Overweight	4	2	2

CG, control group; IG, intervention group; *N,* sample size.

For fidelity of the intervention implementation, the licensed and state-certified teacher completed the log sheet immediately after each lesson. Teacher logs indicated adaptive implementation fidelity during the camp. Although modifications from scripted lesson plans were frequently documented (*M* = 80% ± 40%), These adaptations did not remove the core lesson content and were intended to maintain participation and activity intensity; however, their lesson-specific effects on METs were not assessed. Common modifications included adjusting game rules, adding movement variations, and changing task organization to accommodate camper needs and group dynamics. Modifications were made at the teacher’s discretion when needed. The teacher reported a mean student focus score of 3.20 ± 0.75 on a 5-point scale (1 = far below average, 2 = below average, 3 = average, 4 = above average, and 5 = far above average) and a mean student enjoyment score of 2.40 ± 0.49 on a 3-point scale (1 = not at all, 2 = a little, and 3 = very).

### 3.2 Acceptability of the *ProudMe Tech* mobile app

The campers’ total perceived acceptability showed a modest favorable change from Week 1 to Week 2. The total acceptability percentage score increased from 54.4 ± 21.8 in Week 1 to 58.3 ± 20.3 in Week 2, with a mean difference of 3.9 percentage points and a small-to-moderate paired effect size (*Cohen’s d* = 0.43). Similar patterns were observed across all acceptability factors, with Week 2 percentage scores numerically higher than Week 1 scores. Detailed results are presented in **Table 3**.

**Table 3:** Acceptability of the ProudMe Tech mobile app (n = 13).

Acceptability factor	Week 1 *M ± SD* *(%)*	Week 2 *M ± SD (%)*	Mean difference	*Cohen’s d*
Relative advantage	56.7 ± 18.4	58.5 ± 17.4	1.8	0.18
Simplicity	62.1 ± 22.0	66.7 ± 19.3	4.6	0.29
Trialability	46.2 ± 24.9	50.4 ± 24.3	4.2	0.44
Observability	45.2 ± 25.4	51.0 ± 20.2	5.8	0.42
Translatability	55.4 ± 16.4	60.6 ± 19.8	5.2	0.46
Total acceptability	54.4 ± 21.8	58.3 ± 20.3	3.9	0.43

Percentage scores were calculated as (1 − raw mean/6) × 100, with higher scores indicating greater app acceptability. Week 1 and Week 2 represent assessments at the end of each respective week.

### 3.3 Usage of technology components

#### 3.3.1 Accelerometer wearing

**Figure 2** presents the time and percentage of PA (by intensity) during the 5-hour camp and the nested 2-hour PE for the IG (*n =* 13). During the 5-hour daily camp, participants had 46.6% sedentary behavior, 13% LPA, and 40.4% MVPA (121.3 min/day). During the 2-hour PE segment, participants had 29.7% sedentary behaviors and 56.3% MVPA (67.5 min).

**Figure 2:**
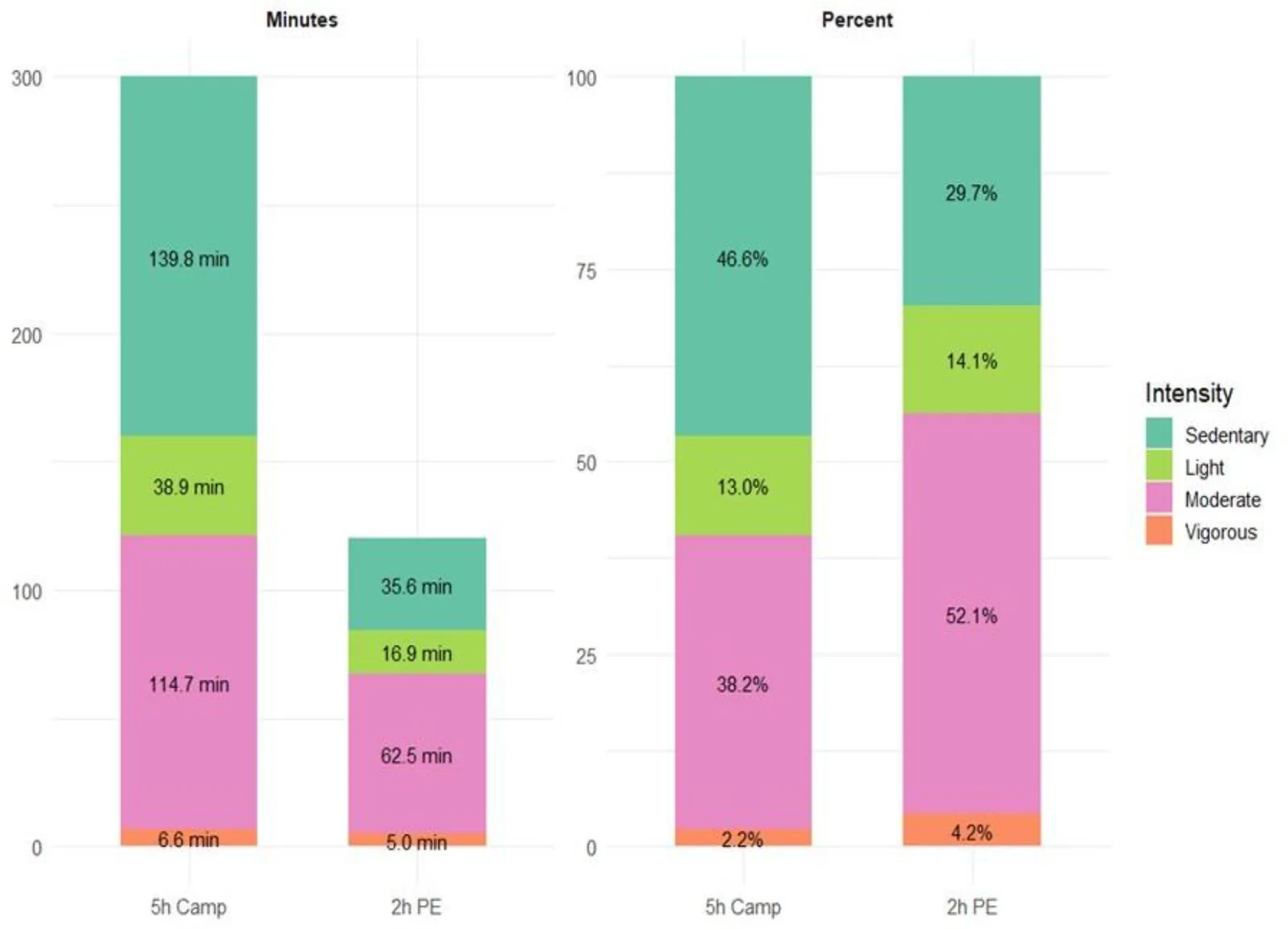
PA intensity distribution: camp day vs PE session (*n*=13).

#### 3.3.2 Fitbit wearing

**Table 4** outlines the usage of Fitbit watches during the intervention period. Only 10 of the 13 intervention participants had usable Fitbit records. The three post records were due to one lost Fitbit, and two participants had unsuccessful device synchronization, resulting in no exportable activity data. The number of dates wearing varied widely among campers.

**Table 4:** Fitbit-reported PA during the intervention.

Item	Value
Reported PA days, range	4–43 days
Participants with records	*n* = 10
Reported PA days	196 person-days; 19.6 ± 14.2 days/participant
Fitbit-recorded PA	30,079 min total; 153.5 min/reported day

**Figure 3** illustrates the amount of daily PA time among the campers who contributed Fitbit data. The shaded area (± *SD*) highlights notable inter-individual differences, yet the group maintained a consistent level of average daily PA. The results indicated lower PA on weekend days than weekdays (135.6 vs. 166.2 minutes per day), with the difference reaching statistical significance (*p =* 0.039).

**Figure 3:**
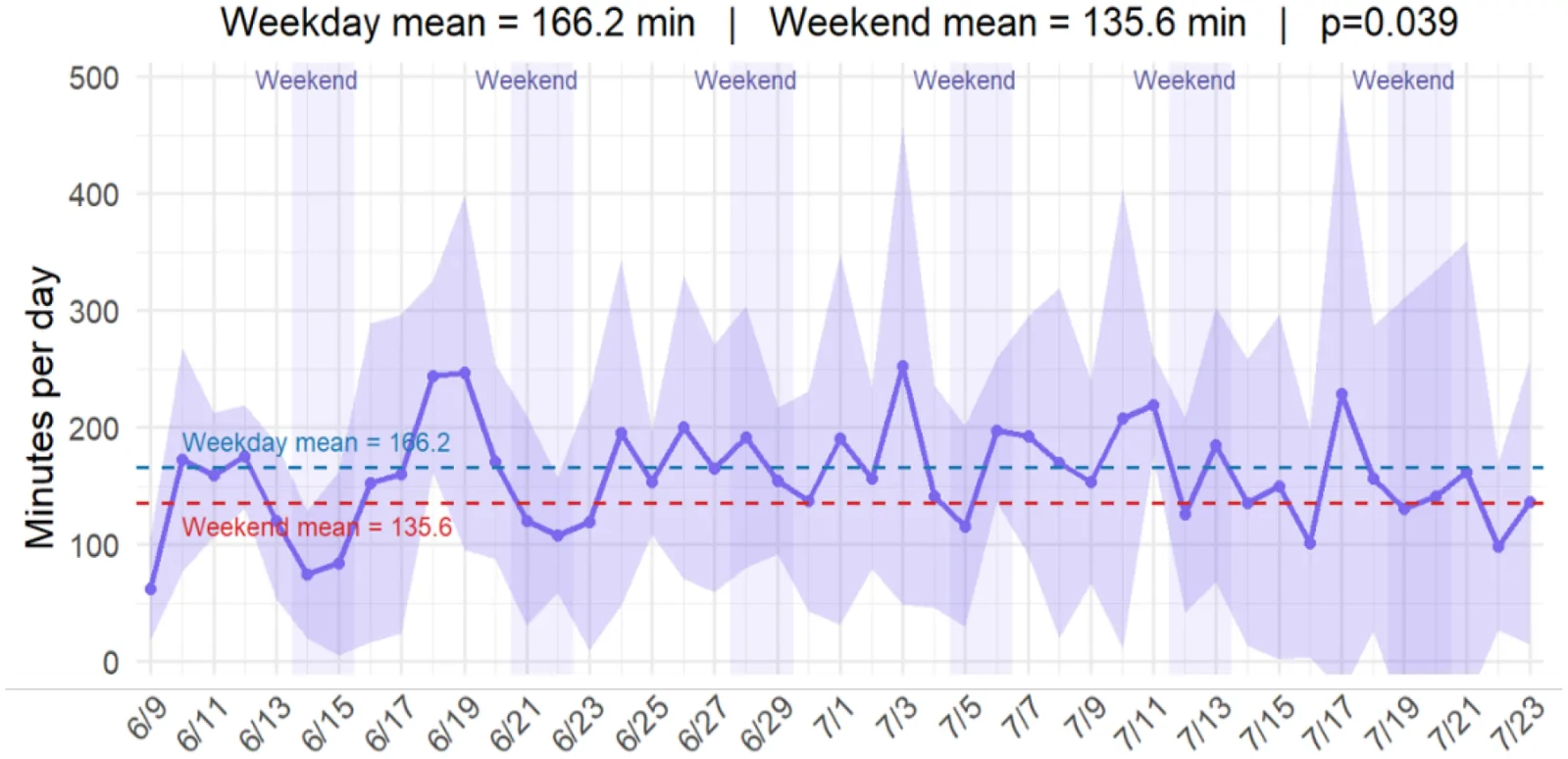
Fitbit daily data.

#### 3.3.3 *ProudMe Tech* mobile app engagement

Across the 8-week summer intervention, participants recorded a total of 851 *ProudMe Tech* mobile app entries. **Figure 4** shows that engagement was high initially in the first two weeks of the in-person camp (271 and 247 entries, respectively) and then steadily declined in subsequent weeks during the post-camp intervention, despite the periodical parent reminders.

**Figure 4:**
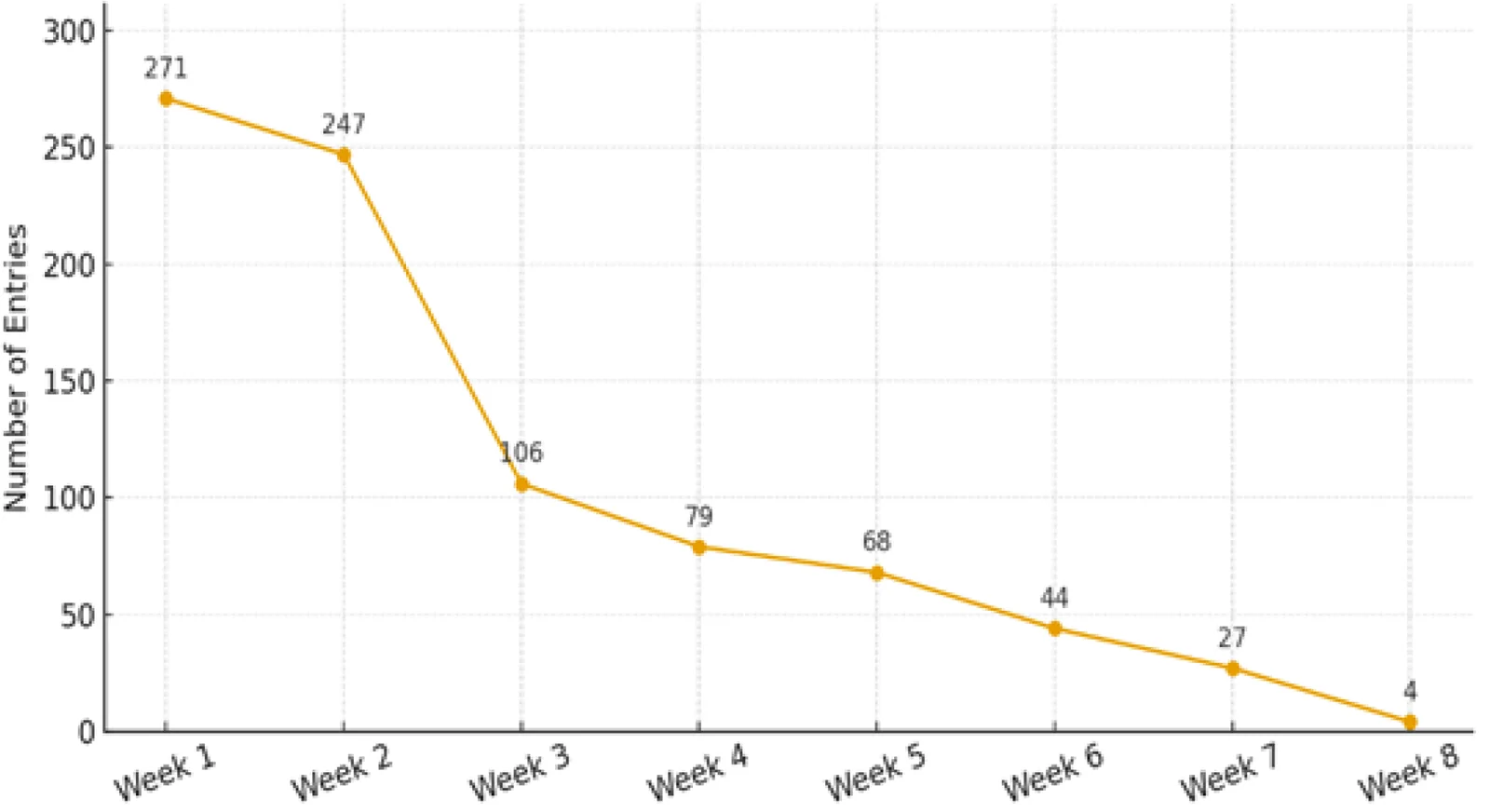
Engagement of app.

In **Figure 5** the left plot presents a total of 851 AI-generated feedback messages categorized into four tone types. *Positive reinforcement* dominated the chatbot output, accounting for 571 messages (67.1%). This was followed by *Educational prompts* totaling 132 messages (15.5%). Only 77 *Constructive suggestions* (9.0%) and 71 “*other*” remarks (8.3%) were observed. The right plot depicts the distribution of feedback per user. *Positive reinforcement* was the most frequent category, averaging 35.7 messages (SD = 23.0) per user, followed by *Educational prompts* (*M* = 8.2, SD = 7.4), *constructive suggestions* (*M* = 4.8, SD = 5.3), and *Other* (*M* = 4.4, SD = 6.3). Overall, the AI chatbot’s feedback was skewed toward affirmation rather than instruction or corrective messaging.

**Figure 5:**
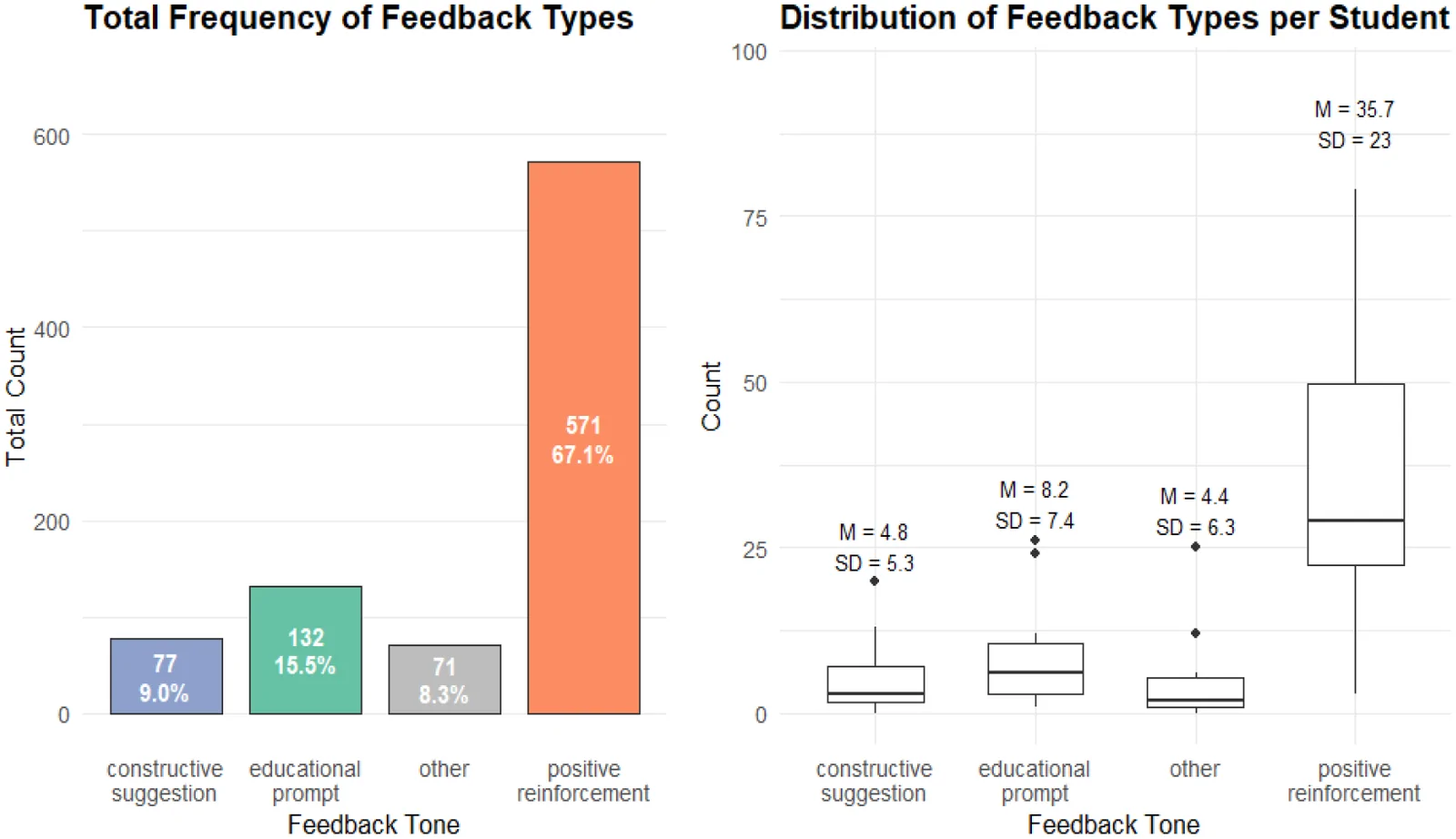
Feedback sentiment vs total feedback and feedback types per student average.

### 3.4 Preliminary effect

#### 3.4.1 Anthropometric outcomes

Anthropometric outcomes, including weight, BMI, and waist circumference for the IG and CG by time of measurement, are illustrated in **Figure 6** (*N* = 25). Figure labels show *M* and within-group paired-comparison *p* values relative to Pre. Both groups showed significant increases in weight from Pre to Post (IG: *p* = .045; CG: *p* = .012), with the IG also showing a significant increase from Pre to Mid (*p* = .037). BMI remained relatively stable in both groups, with no significant pre-to-post changes observed in either the IG (*p* = .774) or CG (*p* = .087) and no significant Pre-to-Mid change in the IG (*p* = .750). For waist circumference, the IG showed a significant decrease from Pre to Mid (*p* = .023), but this reduction was not maintained at Post, when waist circumference increased above the Pre level (*p* = .005). The CG also showed a significant pre-to-post increase in waist circumference (*p* <.001).

**Figure 6:**
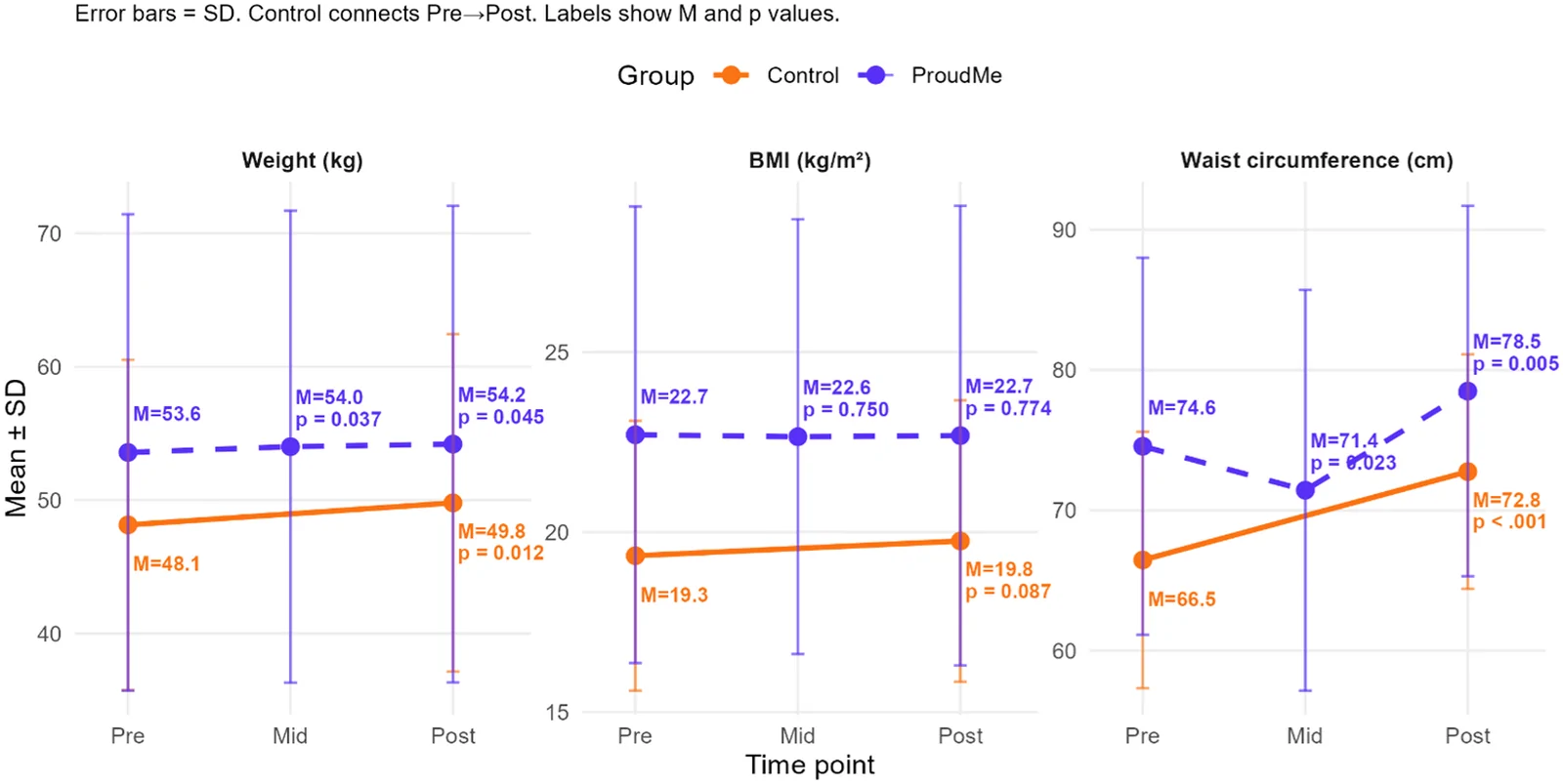
Changes in anthropometric outcomes.

Effect sizes were calculated to describe pre-to-post anthropometric changes. Within-group paired Cohen’s *d* values indicated small changes in weight for the CG (*d* = 0.13, 95% *CI* [0.05, 0.25]) and IG (*d* = 0.03, 95% *CI* [0.01, 0.08]). BMI showed a small change in the CG (*d* = 0.11, 95% *CI* [0.00, 0.23]) and a negligible change in the IG (*d* = -0.004, 95% *CI* [-0.04, 0.02]). Waist circumference showed a larger within-group change in the CG (*d* = 0.72, 95% *CI* [0.52, 1.49]) than in the IG (*d* = 0.30, 95% *CI* [0.13, 0.60]). Between-group pooled Cohen’s *d* values based on change scores were 0.68 for weight, 0.77 for BMI, and 0.73 for waist circumference, respectively, indicating descriptively larger increases in the CG than in the IG. However, these differences should be interpreted cautiously because the group × time interactions were not statistically significant.

#### 3.4.2 Accelerometer-measured PA

**Figure 7** illustrates the PA behaviors by intensity, time, and group. Repeated measures ANOVA showed significant main effects of time for moderate PA, vigorous PA, and total MVPA. Significant group main effects were observed for light PA, moderate PA, and total MVPA, suggesting that the CG had higher overall activity levels than the IG across time points. However, Group × Time interactions were not significant for any PA outcomes.

**Figure 7:**
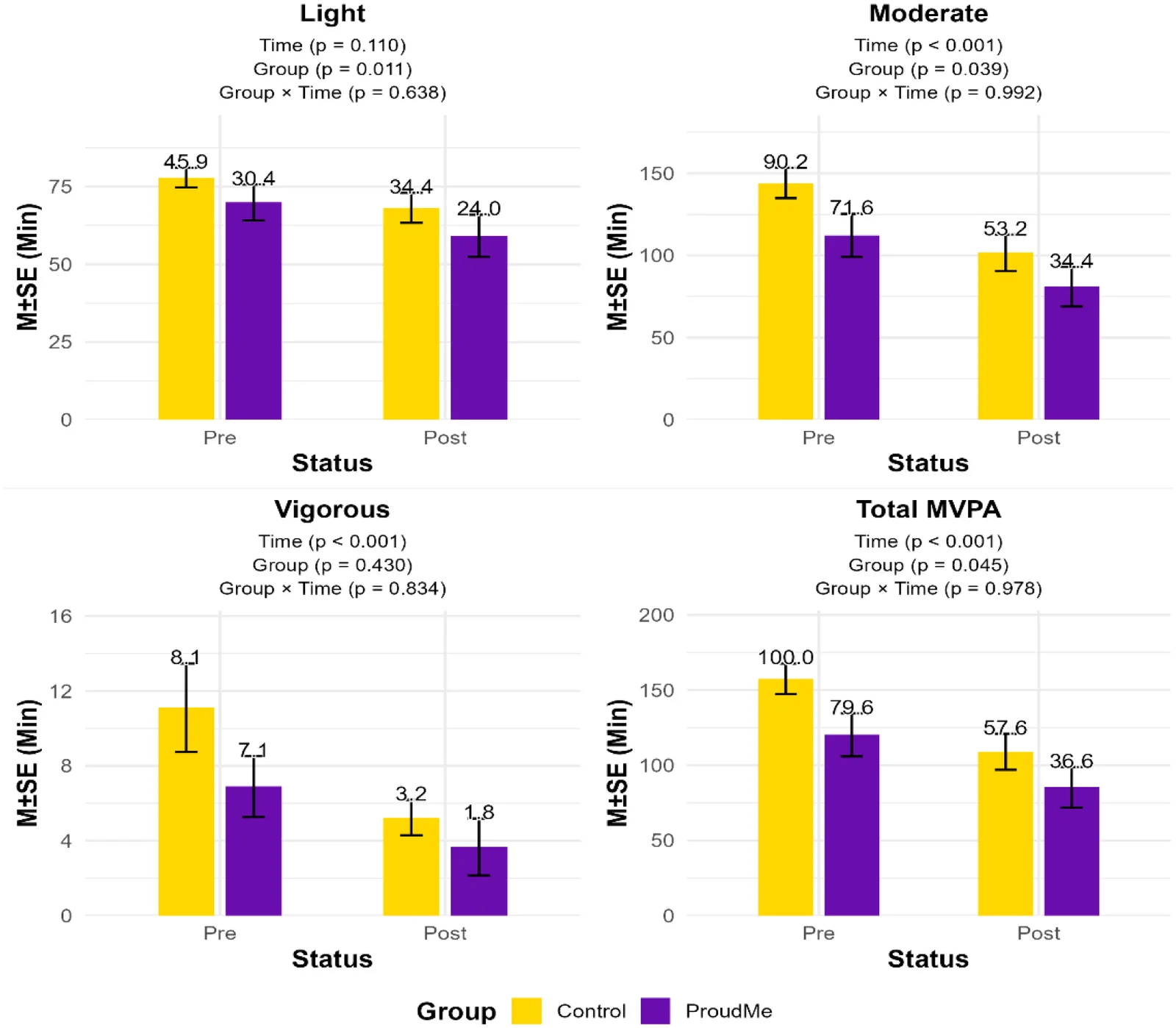
Pre to Post group accelerometer data. Sample sizes reflect participants with valid accelerometer data at each assessment: CG: Pre *n =* 12; post *n =* 10; IG: Pre *n =* 14; Post *n =* 13.

#### 3.4.3 Self-reported health behaviors

**Figure 8** shows the results for PA, fruit/vegetable intake, and screen time by time and group (*N* = 25). Two indicators were used to assess PA (active days and weekly frequency of MVPA). The days meeting ≥60 min MVPA showed an increase pattern in the IG compared to a decline pattern in the CG. For leisure-time MVPA frequency, IG showed a slight decrease in score, suggesting less frequent leisure-time MVPA, whereas the CG showed a slight increase. For fruit/vegetable intake (servings/day), CG remained stable, but IG declined slightly. CG further showed decreased screen time from 9.92 to 9.00, whereas the IG increased from 8.69 to 10.77. Despite these varying change patterns by condition, no statistically significant interaction effects were observed (*p* > 0.05).

**Figure 8:**
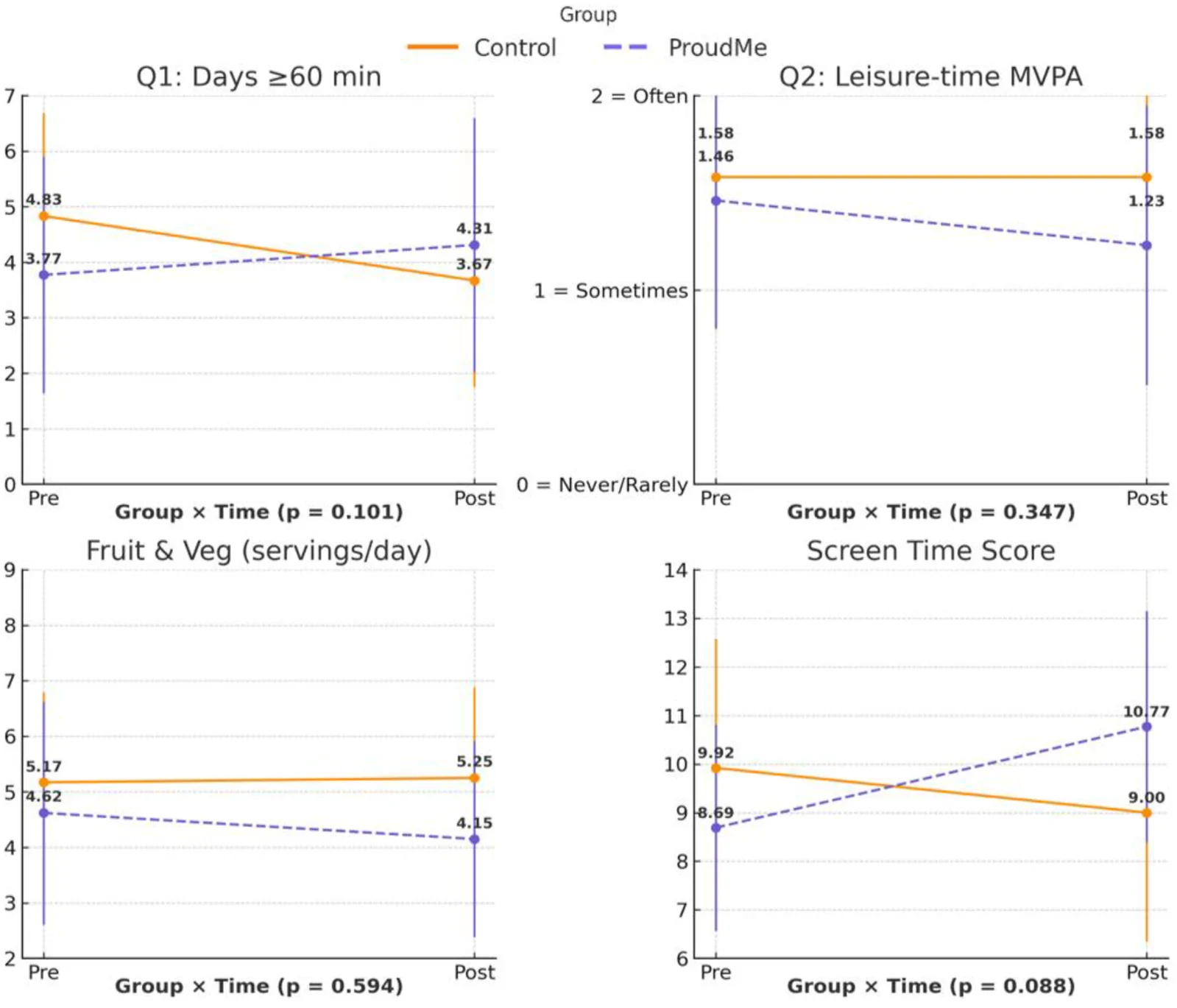
Self-reported health behaviors outcomes.

#### 3.4.4 Self-efficacy and knowledge

**Figure 9** shows that CG demonstrated a slight decline in PA self-efficacy, while the *IG* remained unchanged (*N* = 25), and PA and fitness knowledge increased from pre- to post-test in both groups (CG: from 21.50 to 22.08; IG: from 20.36 to 22.08). No significant interaction effects were observed.

**Figure 9:**
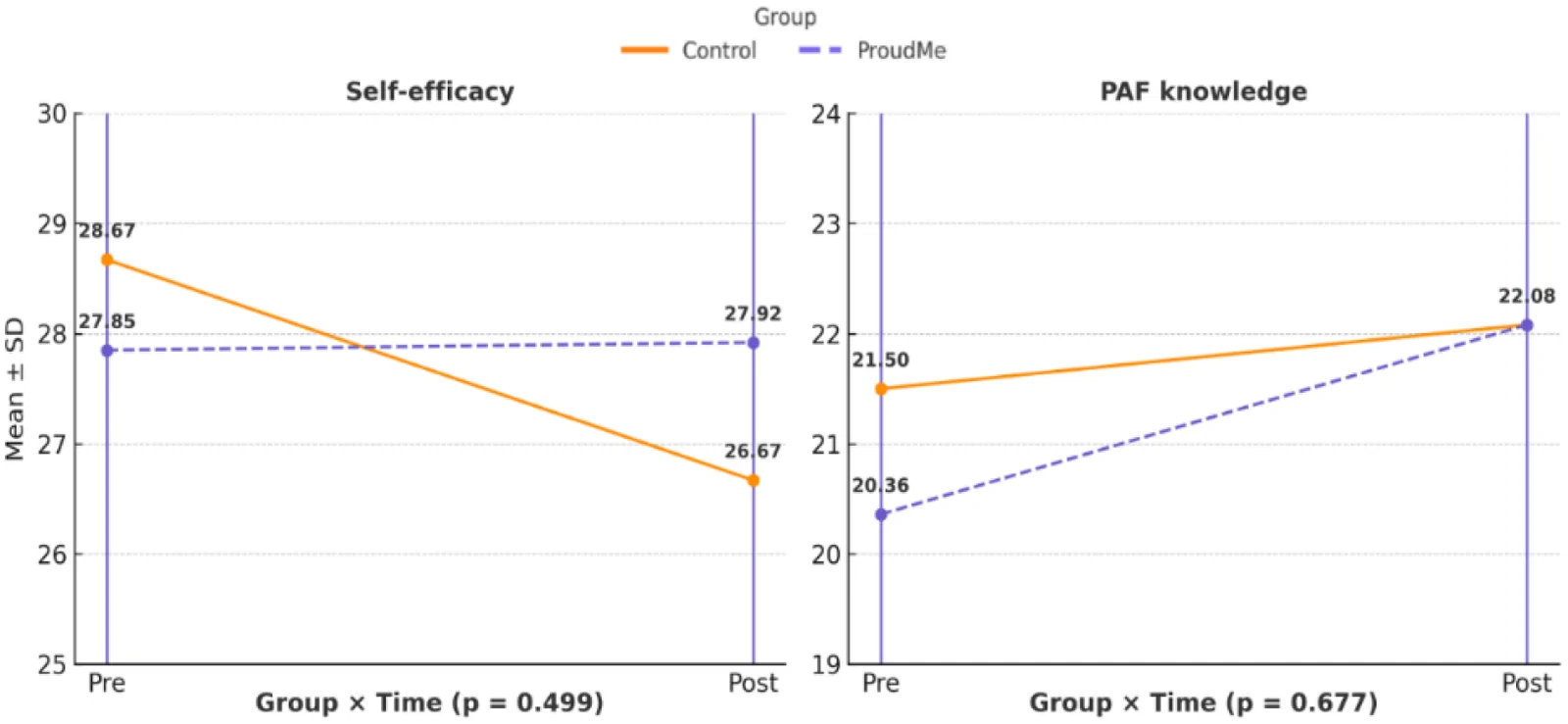
Psychosocial and knowledge outcomes.

## 4 Discussion

### 4.1 Principal findings

This pilot-controlled trial primarily evaluated the acceptability of the *ProudMe Tech* mobile app and the usage of three technology components, while secondarily exploring preliminary anthropometric, behavioral, and psychosocial outcome patterns. In this study, technology-assisted behavioral management referred to app-based goal setting, self-monitoring, behavior reflection, and AI-generated personalized feedback.

Overall, the ProudMe Tech mobile app demonstrated modest acceptability, with total scores increasing numerically from 54.4% in Week 1 to 58.3% in Week 2. Although this pattern suggests a small favorable shift with continued use, the absolute scores remained modest and should not be interpreted as indicating strong acceptability. The mobile app showed high usage initially when the participants were attending the camp on the university campus; however, the usage declined steadily throughout the summer. These patterns are consistent with prior research showing that perceived attributes of eHealth innovations, including relative advantage, simplicity, trialability, observability, and translatability, may shape users’ acceptability and adoption (Atkinson, 2007; Perski et al., 2017). The decline in the mobile app usage over time also reflects the broader attrition challenge commonly observed in eHealth interventions (Eysenbach, 2005). Prior reviews further suggest that intervention characteristics and persuasive design features, such as reminders, feedback, and structured support, may improve adherence to web-based health interventions (Jensen et al., 2016; Kelders et al., 2012; Tan et al., 2026).

The accelerometer data further indicated that the in-person camp and PE sessions were physically engaging, with participants spending a substantial proportion of time in MVPA particularly during the 2-hour PE time. However, usage and sustained engagement varied across technology components. Fitbit data varied widely across participants with wearing time ranging from four to 43 days. During the 6-week extension period, families were encouraged to support participants continued use of the *ProudMe Tech* mobile app 3–5 times per week and daily Fitbit wear. The higher level of PA on weekdays might have reflected biased PA time toward the more compliant participants. The observed higher Fitbit-recorded PA on weekdays may be consistent with the *structured day hypothesis* (Weaver et al., 2020); however, given the small number of participants with usable data records and the wide variation in recorded days, this finding should be interpreted cautiously as a descriptive pattern and suggests the need for future PA interventions on weekend days during summertime. This finding is consistent with the idea that even during summer, weekdays may provide more routine and external structure than weekends (Brazendale et al., 2017). Although Fitbit-supported self-monitoring may have helped participants maintain awareness of their activity, Fitbit data alone did not indicate whether participants used the device to actively regulate their behaviors. During the post-camp extension period, families received parent-facing seminars, reminders, and healthy living resources and were encouraged to support *ProudMe Tech* mobile app use 3–5 times per week and daily Fitbit wear. However, app engagement still declined after the in-person camp, suggesting that general parent-facing reminders and resources may not have been sufficient to sustain adolescent engagement at home. Future studies should consider combining Fitbit monitoring and mobile app use with more structured and individualized engagement supports, such as automated app reminders, missed-entry notifications, scheduled check-ins, parent-child goal review, or coaching prompts, which may better align the intervention with theoretical mechanisms (Ajzen, 1991; Au et al., 2024). Together, these findings show that the technology-assisted behavioral management components could be implemented within the structured summer camp setting, although sustained technology usage during the post-camp extension was limited. More specifically, the camp was physically engaging overall, but sustained engagement with technology-supported self-monitoring after camp remained challenging.

The preliminary effect evidence concerning the summer obesity prevention intervention was mixed and should be interpreted cautiously. Anthropometric outcomes suggested some favorable short-term change patterns during the camp period. Waist circumference decreased from pre-intervention to the end of camp in the intervention group, but this improvement dissipated at post-test. Weight increased in both groups, although the increase was descriptively smaller in the IG. BMI remained relatively stable in IG and showed a small, non-significant increase in the CG. Group × time interactions were not significant. Slight increases in BMI over the summer may partially reflect normal growth among adolescents rather than adverse weight gain, because BMI often increases during puberty alongside changes in height, lean mass, and body composition (Gaml-Sørensen et al., 2025; Sarafoglou et al., 2017). Therefore, the more stable BMI pattern and smaller weight increase observed in the IG should be interpreted as possible attenuation of summer-related anthropometric gain rather than clear evidence of intervention effectiveness. As previous behavioral interventions have shown, initial improvements often wane without sustained reinforcement (Anderson and Hudson, 2022); thus, the absence of extended follow up limits our ability to determine whether the camp’s benefits persisted beyond the structured program period.

Similarly, health behaviors and psychosocial outcomes showed mixed and mostly non-significant patterns. Because both assessments occurred during the same summer period, the decline in MVPA was unlikely to reflect a school-year-to-summer seasonal transition. However, the similar decline in both groups and the absence of significant group × time interactions suggest that it may reflect broader time-related factors (e.g., seasonality) or measurement variability rather than an intervention-specific effect. Notably, self-reported screen time increased in the IG from 8.69 to 10.77, whereas it decreased in the CG from 9.92 to 9.00; however, the group × time interaction was not statistically significant. Because screen time was self-reported and the study did not directly assess participants’ reasons for the observed change. This finding suggests that increasing awareness alone may be insufficient and that stronger family-based monitoring, parental integration, and reinforcement may be needed to help adolescents translate awareness into reduced screen use and other obesogenic behaviors.

However, several outcomes demonstrated favorable numeric trends, suggesting potential benefits of the intervention despite the small sample size. The non-significant findings should therefore be interpreted cautiously, as this pilot study was not adequately powered to detect small intervention effects. These preliminary patterns are consistent with prior summer intervention studies, in which structured programming showed promise for improving children’s anthropometric and behavioral outcomes, although effects were sometimes mixed or modest after the supervised period ended (Evans et al., 2020; Larsen et al., 2014). Notably, self-reported screen time increased in the IG from 8.69 to 10.77, whereas it decreased in the CG from 9.92 to 9.00. The group × time interaction was not statistically significant. More recent randomized evidence also suggests that free summer day camp access can improve children’s MVPA and reduce sedentary time and screen time, supporting the potential value of structured summer programming for obesity prevention (Beets et al., 2025). Although the integration of *ProudMe Tech* mobile app was a novel element of the present intervention, the combination of modest acceptability and declining engagement of the mobile app and Fitbit suggest that technology alone may be insufficient to sustain long-term motivation. Future versions of the summer intervention may need stronger behavioral scaffolding, such as scheduled check-ins, family-based reinforcement, automated reminders, and more personalized feedback loops, to support continued self-monitoring during the post-camp extension period.

### 4.2 Implications for practice

These results underscore the potential of integrating technology-assisted behavioral management into summer camps as viable approach to prevent adolescent obesity. Camps offer a concentrated opportunity to engage adolescents in supervised, multi-component programs that address health behaviors and outcomes (Zeraattalab-Motlagh et al., 2025). However, to extend effects beyond the camp setting, interventions should incorporate technology-assisted reinforcement strategies. Integration of AI coaching and behavior monitoring could strengthen sustainability. However, it is of utmost importance to actively involve and engage parents in these intervention initiatives. In the present study, regular parent reminders and educational resources may not have been sufficient to promote active parental involvement. Future interventions should move beyond parental reminders toward parental integration, such as parent-child shared goals, cooperative physical activity challenges, joint review of app progress, and family-based reinforcement. Further, the two-week camp followed by six weeks’ technology-assisted behavioral management may not be the most effective model to render significant effect. Future interventions may consider increasing camp exposure and alternate in-person camp and remote activities weekly, to create a more conducive intervention schedule and structure during the summer period.

### 4.3 Limitations

Several limitations should be acknowledged when interpreting these findings. First, the small sample size (*N =* 25) substantially limited the statistical power, increasing the likelihood of Type II errors and reducing the precision of effect estimates. Because group allocation was based on participant availability rather than random assignment, selection bias cannot be excluded. The coding program served as a comparison activity intended to match the time and attention associated with app use in the IG condition; however, the two programs differed substantially in structure, supervision, and physical activity opportunities. In addition, the observed differences in racial composition and baseline MVPA may have confounded between-group comparisons. Individual-level socioeconomic status and baseline parental support were not measured; therefore, possible group differences in these factors could not be evaluated. Accordingly, the observed outcome patterns cannot be attributed specifically to the ProudMe intervention or its technology components, and the group-related findings should be interpreted cautiously.

In addition, multiple exploratory pre–post and group × time comparisons were conducted without adjustment for multiplicity, which increased the risk of Type I error. Therefore, statistically significant findings should be considered hypothesis-generating and interpreted cautiously rather than as definitive evidence of intervention effectiveness. These preliminary findings require confirmation in adequately powered trials with prespecified primary outcomes and appropriate multiplicity-control procedures.

Another limitation is that participants were not specifically recruited based on elevated individual-level obesity risk indicators, such as low baseline PA, poor diet quality, excessive screen time, or overweight/obesity status. Eligibility was primarily based on age, geographic proximity, neighborhood-level area deprivation, mobile device access, and transportation availability. Therefore, some participants may have already had relatively favorable baseline health behaviors or anthropometric profiles, leaving limited room for measurable improvement during the short intervention period. This may have attenuated the observed changes in anthropometric, behavioral, and psychosocial outcomes.

Second, the three technology components also differed in data completeness and evidentiary strength. Accelerometer data (for pre and posttests) provided the strongest objective PA evidence but were limited by valid wear time requirements (10-hour per day) and small sample sizes. Fitbit data provided descriptive activity monitoring during the intervention period but was available only for IG participants with usable records and its usage varied widely across participants. Another limitation is that participants’ activities during the 6-week technology-supported extension period were not systematically documented. Some adolescents may have participated in other camps, sports, travel, or structured summer activities after the in-person *ProudMe* Camp ended. *ProudMe Tech* mobile app records captured detailed user engagement, but entries became sparse in later weeks. It is important to distinguish app acceptability from sustained app usage. Acceptability was assessed only during the in-person camp, whereas app entries were used to describe usage across the eight-week intervention. Therefore, the decline in post-camp entries does not demonstrate that initial acceptability was merely a novelty effect. Because reasons for disengagement were not directly assessed, the respective influences of reduced structure, counselor support, parental involvement, competing activities, or novelty cannot be determined. Third, the non-randomized design might have introduced selection bias. However, randomizing participants would be challenging, as not all participants were available to attend the camp. Additionally, the reliance on self-reported diet and screen time measures was not desirable due to social desirability errors. Objective measures would be more accurate, but they also have limitations. Finally, this pilot-controlled trial was not prospectively registered, which limits protocol transparency. Our future fully powered trial would be prospectively registered before participant enrollment.

### 4.4 Future directions

Future studies should test this hybrid model in larger randomized controlled trials with longer follow-up periods. Given recent evidence that extended summer day camp access can improve children’s MVPA and reduce sedentary time and screen time, future research should examine whether technology-assisted summer obesity prevention interventions can produce similar or greater effects when delivered with longer exposure, stronger post-camp support, and greater parental engagement (Beets et al., 2025). Additional strategies, such as increasing camp exposure, alternating in-person camp sessions with remote technology-supported activities, and strengthening parental involvement, may help sustain engagement and strengthen intervention effect.

## Data Availability

The datasets generated and analyzed during this study are not publicly available due to confidentiality concerns and ethical restrictions. De-identified data may be made available from the corresponding author on reasonable request and after approval by the Institutional Review Board. Requests to access the datasets should be directed to SC, senlinchen@lsu.edu.
